# Supplementary material for: Acceptance and compliance with micronutrient powder (MNP) among children aged 6–23 months in northern Nigeria
Source: PLOS Glob Public Health. 2022 Oct 17;2(10):e0000961. doi: 10.1371/journal.pgph.0000961 (PMC10022258; doi:10.1371/journal.pgph.0000961)
Supplement: S1 Dataset — (ZIP) [file pgph.0000961.s008.zip › S8_Dataset/Spot Checks.zip!/Spot Checks/08072015_A_GA_AS_2_SC.pdf]

SPOT CHECK RECORDING FORM

State: Adamawa _ LGA:_ganye__________ Community: ganye1_

Name of caregiver (female): …lare hassan…………….

Name of caregiver (male): ……Hassan adamu mohammend…………………._

Sex of index child: female_____ Age of index child (in months): 21month________

Spot Check Information

1. **Date of MNP distribution to household** (circle option below based on LGA name above)**:**
   - **Kebbi:** BirninKebbi&Jega LGA: **June 16**
   - **Kebbi:** Danko-wasagu: **June 17**
   - **Adamawa:**Song, Ganye: **June 18**
   - **Adamawa:**Guyuk **June 19**
2. **Date of Spot Check Observation: _08/07/2015________________**
3. **Number of sachets remaining**(Ask the caregiver to show you and count the specific number of sachets remaining)**:**

   39

4. **Number of sachets that should remainusing one MNP sachet per day** (Analyst only)**:**

   41

5. **Number of sachets remaining / number of sachets should be remaining**(Analyst only)**:**

   ______39_____ / _____41______ = _____95.1______ %

Probes

Question 1. You have **XX** MNP sachets remaining. Explain how easy or difficult it was to use the MNP product on a daily basis for your child 6 – 23 months? (Circle one answer)

Very Difficult **Difficult** **Easy** **Very Easy**

Question 2. Describe any specific challenges that you faced that made the MNP difficult to use on a daily basis?

There are no challenges in general but it is just that the MNP sachet is too thick and difficult to used daily.

Question 3. Describe specific aspects of the MNP product that you or your child enjoyed that made it easier to use on a daily basis?

The aspect of the product is that i enjoy the most is the fact that it looks like powder milk and does not show any bubbles in the kunu and looks as if nothing was added into it.

Question 4. What questions do you have about the MNP based on your experience using it?

When we start administering the product and noticed that the child has fever or running temperature, what do we do about that.

Question 5. What specific recommendations do you have that we can do as a program to make the MNP easier to use on a daily basis?

The product is too thick, I mean the leather of the product or the sachet should be made a little lighter for easy day to day use because most of us use razor blade to unseal it. That is the only recommendation. Every other thing is just okay and we are so grateful.
